# Quality Improvement Projects and Anesthesiology Graduate Medical Education: A Systematic Review

**DOI:** 10.7759/cureus.57908

**Published:** 2024-04-09

**Authors:** James H Jones, Neal Fleming

**Affiliations:** 1 Anesthesiology, University of North Carolina at Chapel Hill, Chapel Hill, USA; 2 Anesthesia, UC Davis Medical Center, Sacramento, USA

**Keywords:** monte carlo simulation, academic anesthesiology, systematic review, graduate medical education, quality improvement

## Abstract

Quality improvement (QI) projects are essential components of graduate medical education and healthcare organizations to improve patient outcomes. We systematically reviewed the literature on QI projects in anesthesiology graduate medical education programs to assess whether these projects are leading to publications. A literature search was conducted in July 2023, using PubMed, Embase, and the Central Register of Controlled Trials (CENTRAL) for articles describing QI initiatives originating within the United States and applicable to anesthesiology residency training programs. The following data were collected: intervention(s), sample size (number of participants or events), outcome metric(s), result(s), and conclusion(s). One hundred and fifty publications were identified, and 31 articles met the inclusion criteria. A total of 2,259 residents and 72,889 events were included in this review. Educational modalities, such as simulation, training sessions, or online curricula, were the most prevalent interventions in the included studies. Pre-intervention and post-intervention assessments were the most common outcome metrics reported. Our review of the literature demonstrates that few QI projects performed within anesthesiology training programs lead to published manuscripts. Further research should aim at increasing the impact of required QI projects within the sponsoring institution and specialty.

## Introduction and background

The Accreditation Council for Graduate Medical Education (ACGME) establishes Common Program Requirements, or shared goals, for all specialty training programs. Since the addition of quality improvement (QI) projects to these requirements in 2012, residents have been expected to meet the following objectives upon graduation: demonstrate competency in analyzing the quality of care they provide, achieve organizational patient safety goals, prioritize activities and implement changes with the goal of care improvement, and evaluate the impact of interventions [1]. In 2018, the Clinical Learning Environment Review (CLER) Program highlighted the underrepresentation of trainees on organizational QI committees and a discrepancy between resident projects and organizational quality of care goals [2].

The reasons for lack of resident engagement with organizational quality initiatives are likely multifactorial. Traditional QI methods, such as statistical process control (SPC) charts, require substantial time to collect data and properly evaluate an intervention. Still, these methods may not achieve organizational goals or generate positive results. Furthermore, because QI projects may focus on decreasing the incidence of rare events, collecting sufficient data may preclude completion of projects within the time constraints of residency training.

To evaluate relevant QI projects for United States anesthesiology trainees from the time of the ACGME common program requirement change, we conducted a systematic review of the literature. Our objective was to answer the following research question: In the United States between 2012 and 2022, what are the characteristics of QI projects that have progressed to publication? This review was not registered.

## Review

Methods

Search Strategy

PubMed, Embase, and the Central Register of Controlled Trials (CENTRAL) were searched in July 2023, with the following query: ("medical education," OR "residency," OR "fellowship," OR "medical training") AND ("quality improvement" OR "QI") AND ("curriculum" OR "projects" OR "training" OR "workshop" OR "session" OR "activities" OR "syllabus" OR "modules") AND ("anesthesia" OR "anesthesiology").

Eligibility Criteria

Articles written in the English language in the last 10 years with an available full text and abstract were used. We selected a timeframe of the last 10 years (2012-2022) because the ACGME began requiring resident participation in QI projects in 2012. Articles were excluded if the primary outcome was not applicable to quality improvement or anesthesiology. Studies conducted by departments other than anesthesiology were included if the authors (JJ and NF) deemed them applicable to anesthesiology, such as otolaryngology, trauma surgery, emergency medicine, and critical care. Articles pertaining only to fellowship training were excluded. Review articles, meta-analyses, special articles, editorials, practice guidelines, and commentaries were also excluded, as well as all studies that took place outside of the United States.

Study Selection and Data Collection

The authors (JJ and NF) independently reviewed and extracted the following data from the eligible articles: intervention(s), sample size (number of participants or events), outcome metric(s), result(s), and conclusion(s). Disagreements were resolved with further discussion among the authors until a consensus was reached. Few studies commented on the medical education curriculum, itself, thus precluding appraisal with the Medical Education Research Study Quality Instrument (MERSQI).

Results

Study Selection

**Figure 1:**
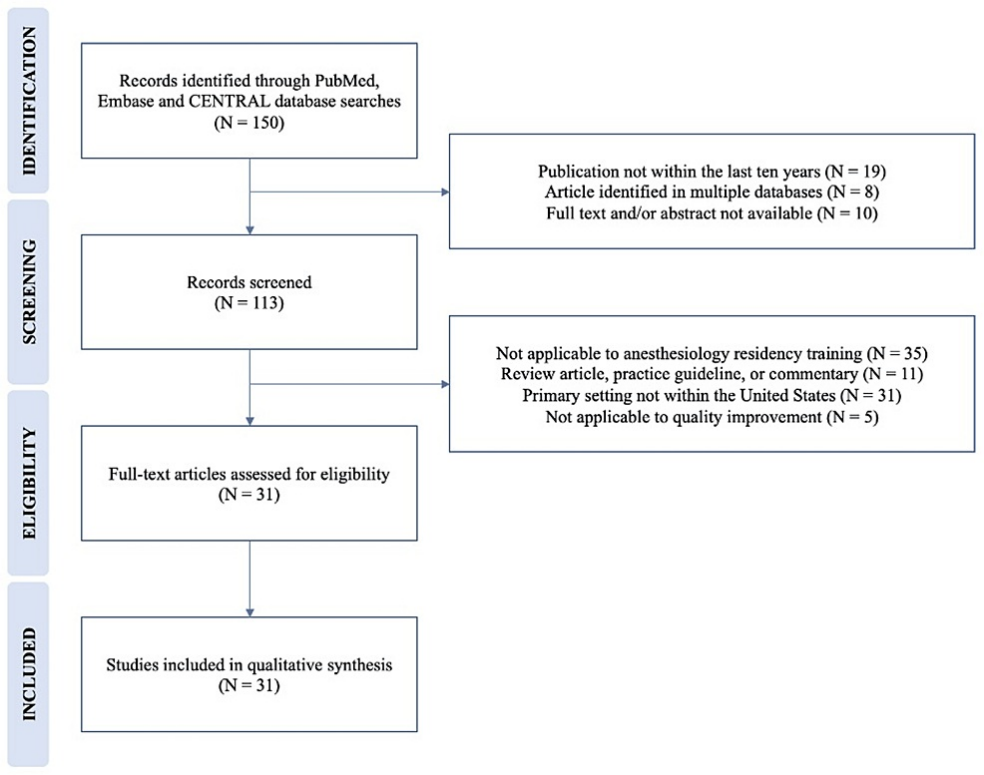
Identification and screening of publications

A literature search in the PubMed, Embase, and CENTRAL databases identified 150 articles. Articles were screened for publication in English in the last 10 years and the availability of an abstract and full text. A total of 119 articles were excluded from this review for the following reasons: publication not within the last 10 years; article identified in multiple databases; full text or abstract not available; not applicable to anesthesiology; not applicable to quality improvement; primary setting not within the United States; and review articles, practice guidelines, special articles, editorials, or commentaries. Figure 1 displays the identification and screening of publications.

Population of Included Studies

A total of 31 studies were identified for data extraction, which included 2,259 residents and 72,889 events (variable of primary interest) [3-33]. Residents accounted for, at least, a portion of the research or study team in 48.39% (15/31) of the studies [3,5-11,13,15,16,18-21,23,24,28,30]. Other represented participants included anesthesiology attending faculty, as well as staff and trainees from otolaryngology, trauma surgery, emergency medicine, intensive care unit (ICU) nursing, nurse anesthesia students, nurses, and respiratory therapy [5,10,21,27,29]. Additional data regarding the post-graduate years of residents were inconsistently reported, and the study participants were, on occasion, nonspecifically identified as “physicians”; thus, it was unclear how many attendings or residents were included among the participants. In the studies that included other professionals, residents were the most represented group at 42.94%, 65.52%, and 71.79% of participants [5,10,21]. We allowed the inclusion of other specialties and professional groups because QI projects commonly involve multidisciplinary teams.

Characteristics of Included Studies

Education was the most common intervention in the included studies [4-6,8,10-12,21,23,26-28,30,31,33]. Of the 2,259 residents included in this review, 36.52% (825/2259) received education in an attempt to achieve the projects’ aims [5,6,8,11,21,23,28,30]. Educational modalities included simulation, training sessions, problem-based learning discussion (PBLD), novel residency rotation, digitalization of lectures, formal teaching, bedside teaching, and online curricula [4-6,8,10-12,21,23,28,30,31]. The most common outcome metrics utilized were surveys or questionnaires and knowledge assessments conducted before and after the intervention [5-8,10,11,13,15,23,24,26,29,30]. Interventions that address common organizational goals were less prevalent, such as improved clinic and operating room (OR) efficiency, decreased postoperative opioid consumption, and sustained increases in hand hygiene compliance [4,14,17,18]. Table 1 displays the characteristics of the included studies. The substantial heterogeneity of the results precluded the meta-analysis.

**Table 1:** Characteristics of the included studies

Study (First author)	Intervention(s), observation(s), or initiative(s)	Sample size	Outcome metric(s)	Selected result(s)	Conclusion(s)
Yadav [32]	Modified cardiovascular component of the Sequential Organ Failure Assessment	16,386 ICU admissions	ICU mortality; 28-day mortality	Improved prediction of ICU mortality (0.836 vs. 0.822, p < 0.001); improved prediction of in-hospital mortality (0.799 vs. 0.784, p < 0.001); improved prediction of 28-day mortality (0.798 vs. 0.783, p < 0.001)	Mortality for critically ill patients is better predicted with a modified cardiovascular component to the Sequential Organ Failure Assessment.
Davis [31]	Advanced Resuscitation Training (ART) program	556 cardiac arrests	Arrest incidence, survival-to-hospital discharge, arrest-related deaths, and in-hospital mortality	Decreased arrest incidence (2.7 to 1.2 per 1,000 patient discharges from non-ICU areas); improved survival-to-hospital discharge from 21 to 45% (p < 0.01); improved odds ratio for survival-to-discharge (OR 2.2 95% CI 1.4 to 3.4) and good neurological outcomes (OR 3.0 95% CI 1.7 to 5.3); decreased arrest-related deaths (2.1 to 0.5 per 1,000 patient discharge from non-ICU areas and 1.5 to 1.3 for ICU areas); decreased in-hospital mortality (2.2% to 1.8%)	Resuscitation training program can improve patient outcomes.
Ninan [33]	Didactic program	Not specified	Self-assessment survey of curriculum vitae resume knowledge, personal brand, interview skills, networking, negotiations, practice valuation, benefits analysis, med staff structure and governance, healthcare reform, and confidence in job-finding skills	Improvement in resident-perceived knowledge in all areas measured	Didactic programs can improve career development and leadership skills for anesthesiology residents.
Becker [30]	Daily bedside teaching, examinations, goal communication, topic recording, and “tip sheets”	193 survey responses (168 post-intervention)	Bedside teaching frequency, perceived time at bedside, rounding satisfaction, and rounding efficiency	Increased bedside teaching (10% to 61%); increased perceived time at bedside (37% to 59%); rounding satisfaction (6.7/10 to 7.4/10); no impact on rounding efficiency	Initiatives can improve education during rounds without sacrificing efficiency.
Nett [22]	Database search	10,510 intubations	Adverse events from intubation and procedural details	Desaturation is less common in international PICUs compared to North America (13% vs. 17%, p = 0.001); occurrence of adverse intubation events is lower for international PICUs compared to North America (11% vs 14%, p = 0.003); less cuffed endotracheal tubes (ETT) used internationally (52% vs. 95%, p < 0.001) and inversely correlated with rate of ETT exchange (p < 0.001)	Adverse intubation events are higher in North American PICUs compared to international PICUs.
Veenstra [21]	Advanced surgical airway curriculum	56 general surgery residents and 22 student nurse anesthetists	Test, checklist, and questionnaire. Scores ranged from 1 (would not allow to perform procedure independently) to 5 (would allow to perform procedure independently).	Needle cricothyroidotomy: 5/5 for surgical residents and 4.86 for student nurse anesthetists. Open cricothyroidotomy: ranges from 4.75 to 5 for surgical residents and 4.72 for SRNAs.	Simulation can teach the cognitive and procedural skills necessary to perform needle and open cricothyroidotomy.
Martinelli [23]	Creation of academic medicine rotation during anesthesiology intern year	10 anesthesiology interns	Survey (five-point Likert scale)	Improved confidence in plan–do–study–act (PDSA) cycles (2.5; 95% CI 2.1 to 2.9) and QI projects (2.4; 95% CI 1.9 to 2.9)	An academic medicine rotation improves resident confidence in the appraisal of literature, QI, professional development, and teaching.
Ferraro [24]	Resident chief of QI and patient safety (PS)	28 medicine interns and 49 medicine residents	Survey (six-point Likert scale ranging from medical student at 1, proficiency of clinical faculty at 4, and senior faculty scholar at 6)	Improved resident participation in QI and PS committees (1.5 to 2.7); improved ability to identify system errors (1.9 to 2.9 for residents)	Residency-appointed chief of QI and PS improves resident participation and education with ACGME CLER focus areas.
Galvagno [25]	Record review	1,008 patients	Compliance with quality assurance metrics	13% of patients with hypoxemia received no intervention.	QI assessment directs attention to deficient areas.
Raty [26]	Online modules, lectures, small group sessions	748 students	Course evaluations	The value of small group discussions, course quality, and effectiveness of resident teaching significantly improved (p < 0.05) with time of course and resident involvement.	Resident facilitators improve medical student course quality.
Shao [27]	Online curriculum, checklist, and simulation session	18 (16 physicians and two nurses)	Percentage of completed tasks	Tasks completed increased from 60.3% to 81.8% (p = 0.002).	Training improves physician confidence and simulated critical care scenario performance.
Foong [28]	Compulsory acute pain medicine rotation, summary of workflow, and digitalization of lectures	48 CA-1 residents	Monthly competency scores	Monthly competency scores increased following the implementation of each intervention from 33% to 57%; 60 to 75% and sustained at 75%, respectively.	Fishbone diagrams and Pareto charts can direct medical education improvements.
Shoultz [29]	20-question survey	151 providers (17 anesthesia residents or attendings)	Survey	2% use standardized tools to assess frailty; 37% believe that frailty affects all parts of patients’ health; 87% believe that frailty increases the chance of death.	There is a variable understanding of the definition of frailty and the rare use of standardized assessment tools, despite its perceived importance to patient outcomes.
Chu [3]	Weighted point system for equitable shift distribution	24 residents	Surveys	Reduced overall variance (2016: 63% ± 4.9%, p <0.01; 2017: 57% ± p <0.01)	Work distribution equity is achievable with a weighted point system.
Pimentel [4]	Posters, reminder cards, feedback, and simulation	1,122 events	Compliance	Nonrandom shift and trend: 68% (95% CI 65-72%) to 79% (95% CI 76-83%), p < 0.01	Interventions can lead to sustained increases in hand hygiene compliance.
Kristobak [5]	Problem-based learning discussion (PBLD)	10 attendings and 19 residents	Surveys (five-point Likert scale)	Residents reported the PBLD to be a valuable experience (3.9 ± 0.6). Increased confidence to lead a QI initiative (3.7 ± 0.9). Increased likelihood to start personal QI initiative (3.1 ± 0.9).	PBLD is a feasible method for a QI curriculum.
Tamaki [6]	Formal teaching	168 residents	Pre- and post-intervention quizzes	34.2% improvement (2.7 points, p < 0.001)	Formal education improves tracheostomy knowledge.
Harrington [7]	SICU rotation	98 residents	Pre- and post-rotation surveys	Correlation between communication and experience providing end-of-life (EOL) care	Resident comfort with EOL communication and care is correlated with completion of SICU rotation.
Ziemba [8]	Simulation	289 residents	Pre- and post-simulation assessments	Ability to correctly identify factors required for a root cause analysis (RCA) (62% pre vs. 80% post, p = 0.02). Increase in the intent to ‘always report’ for each adverse event category (3% pre vs. 37% post, p < 0.001).	Simulation is an effective method to teach the components of RCA.
Scales [9]	Specialty-based team competition	422 residents	Percentage of questions attempted and engagement (response time)	Increased questions attempted (79% vs. 68%, p = 0.03) and faster response time (p = 0.006)	Team competition increases resident participation in an online course delivering QI content.
Tsai [10]	Simulation	177 participants	Pre- and post-simulation questionnaires	Improved self-rated team participation, confidence, and knowledge	Simulation can improve team dynamics within an emergency airway response team and individual confidence and knowledge.
Kuza [11]	Training session	42 trainees	Pre- and post-session examination and practical assessments	TTE-naïve mean score improvements with multiple choice questions: 28.2 ± 11.6; and with clinical assessments: 48.6 ± 23.4	A short didactic session on TTE can teach basic skills and encourage its use.
Ramsingh [12]	Online curriculum	686 exams	Diagnostic accuracy of portable point-of-care ultrasound (P-POCUS) compared to traditional assessments and formal diagnostic studies	Higher sensitivity for new diagnoses (p < 0.0001)	An online curriculum can help develop a P-POCUS service.
Flanagan [13]	Electronic tool	654 trainees	Pre- and post-intervention surveys; electronic data indicating problem list updating	Increased problem list updating (p = 0.002) and increased mean new problems added per day (64 pre vs 125 post, p <0.001)	Engagement of house staff in institutional goals is possible with electronic tools.
Cerfolio [14]	Lean and value stream mapping	42 cases	Turnover time	OR turnover time decreased from 37mins to 14mins (p <0.0001); estimated return on investment: $19,500/day	Non-valued steps in OR turnover can be identified with lean and value stream mapping.
Olson [15]	Badges	159 residents	Surveys	Decreased role misidentification (50.8% pre vs 10.2% post, p <0.001) Less gender bias among female residents (65.2% pre vs 31.8% post, p <0.001) Less misidentification from patients among underrepresented residents (84.6% pre vs 23.1% post, p = 0.008)	Role ID badges decrease role misidentification and gender bias.
Cattano [16]	Assessment form	8075 cases	Prediction of difficult airway	Improved predicted rates over time (p = 0.031)	Comprehensive airway assessments did not improve residents' ability to predict a difficult airway, but did improve over time.
Bryskin [17]	Sub-paraspinal block	10 patients	Opioid consumption; functional performance ability	Trend to decreased hydromorphone consumption (24hrs: 0.19 mg/kg vs 0.13 mg/kg, p = 0.72; 48hrs: 0.37 mg/kg vs 0.3 mg/kg, p = 0.37) Improved functional performance ability (POD1: 6.7 vs 4.8, p = 0.0495; POD2: 8.9 vs 6.5, p = 0.04)	Sub-paraspinal block may be a reasonable component of multimodal analgesia.
Williams [18]	Assignment of cases to trainees the day before the patient clinic visit	504 visits	Wait time and session time	Mean wait time: 36.1min vs 21.4min, p <0.01; mean session time: 275.6min vs 247.5min, p <0.01	Case assignments the day prior to clinic visits improve efficiency.
Orebaugh [19]	Combined ultrasound/nerve stimulator blocks	9062 blocks	Incidence of nerve injury and local anesthetic systemic toxicity	Nerve injury 6-12 months: 3 vs 1, p = 0.003 Nerve injury >12 months: 1 vs 0, p = 0.24; seizure: 1 vs 0, p = 0.24	Nerve blocks are safe when performed by trainees with using ultrasound and nerve stimulator.
Epstein [10]	Evaluation by an anesthesia resident the day prior to scheduled inpatient procedure	24735 cases	Surgery cancellation	Most canceled cases were evaluated the day prior to surgery Total canceled minutes: 67.6% (95% CI 64.4%-70.8%, p <10^-6^	More preoperative anesthesia visits are not an economically useful focus for the Perioperative Surgical Home.

Risk of Bias

**Figure 2:**
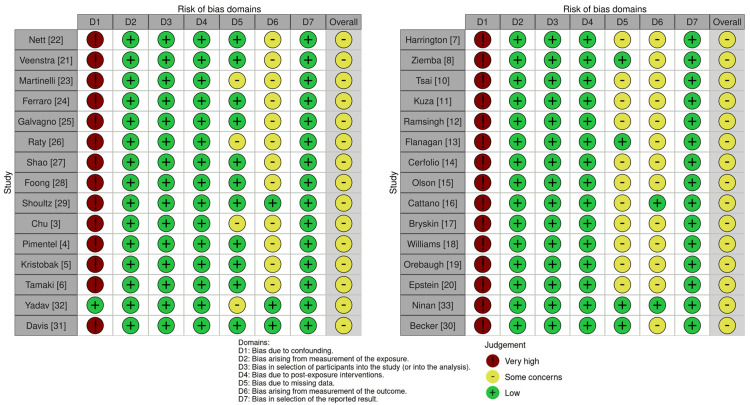
Traffic-light plot for the risk of bias of non-randomized studies

**Figure 3:**
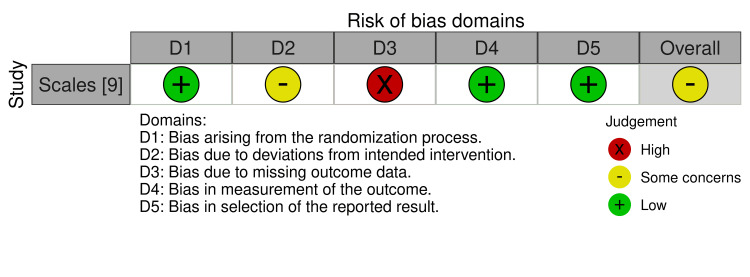
Traffic-light plot for the risk of bias of randomized controlled study

**Figure 4:**
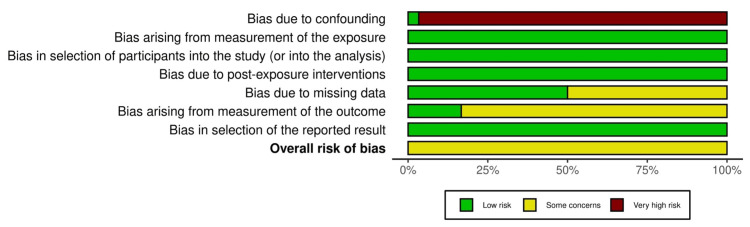
Overall risk of bias of non-randomized studies

Most non-randomized studies demonstrated some risk for bias as measured with the Risk Of Bias In Non-randomized Studies-of Exposure (ROBINS-E) tool from confounding as demonstrated in the traffic-light plot shown in Figure 2 [34,35]. The risk of bias for the only randomized controlled trial was assessed with the risk-of-bias tool for randomized trials (ROB-2) tool, as shown in Figure 3 [34,36]. The randomized-controlled design leads to a low risk for bias due to confounding. The risk of bias due to missing data was common among all studies. The overall risk of bias was rated as 'some concern' for each of the included studies as presented in Figure 4 [34].

Discussion

According to the Association of American Medical Colleges (AAMC), there were 6,371 anesthesiology residents in 2021-2022 [37]. Therefore, a large number of resident QI projects presumably do not progress to publication. This may serve as additional evidence of trainee underrepresentation in organizational quality of care goals as published by CLER [2].

Projects to improve knowledge are important components of residency training and education was the most represented intervention in the publications identified. These projects are relatively straightforward to design and complete and do not require complex statistical analyses. However, because these projects are commonly evaluated with surveys and questionnaires taken by providers, they are unlikely to be impactful with respect to broad organizational quality improvement goals, which are measured with patient outcome metrics. For anesthesiology departments to consistently provide high-quality patient care, educational interventions are required, but these projects, by themselves, are unlikely to produce objective, measurable improvements that healthcare organizations commonly desire. If the intent of the Common Program Requirements is to prepare trainees to grow as leaders in their respective healthcare systems, current efforts may not be consistent with that expectation.

A few published QI projects aligned with common organizational QI goals for patient care, OR and clinic efficiency and hand hygiene [4,14,17,18]. QI projects in support of organizational goals are more likely to require lengthy data collection protocols, and traditional analysis methods may not sufficiently evaluate these goals within the time constraints of residency training. Substantial projects can be passed on between graduating resident classes, yet this practice may deprive residents of valuable experiences gained from attempting their own quality initiatives.

This review of the literature characterizes the quality improvement projects that have been published by departments with GME curricula in the United States since the inclusion of QI in the ACGME Common Program Requirements in 2012. Each project has been described with respect to the key participants of the project and the interventions and outcomes. These data highlight that published QI projects performed within United States departments with GME curricula tend to focus on educational interventions and usually do not comment on their respective organization’s QI goals. 

Limitations

The authors recognize several limitations in this systematic review. The search query used to identify articles may not be inclusive of all relevant studies. For example, projects conducted outside of the United States may be relevant to general anesthesiology quality initiatives, particularly those that originate in Canada, which has similar graduate medical education requirements. We decided against the inclusion of projects conducted outside of the United States in an effort to best characterize the impact of QI’s inclusion to the Common Program Requirements by the ACGME (an organization that governs GME programs within the United States) in 2012.

PubMed, Embase and CENTRAL may not sufficiently account for all relevant studies. We did not include meta-analyses, systematic reviews or Cochrane reviews in our database searches. Although the inclusion of such publications may have identified additional relevant studies, we sought only primary research so that our search strategy could be replicated and to eliminate the subjective inclusion of studies referenced in other reviews. Again, not reviewing the references from other reviews may have limited the number of relevant articles identified. However, we suspect this objective search strategy in this study’s design allows for replication.

Most non-randomized studies demonstrate a high risk of bias due to confounding, which is likely related to the observational design of many projects. All studies demonstrated 'some concern' for the risk of bias, thus serving as a limitation for qualitative synthesis of the results.

The absence of publications is not interchangeable with the absence of work. While there is a dearth of publications highlighting resident quality improvement projects, this should not be confused with a lack of high-quality work and may alternatively suggest that publications are not being submitted for other reasons (such as lack of novelty of the project or negative result).

Future Research

Future research should aim to enhance the impact of quality improvement projects. Statistics software has been used previously to simulate the results of quality initiatives prior to their implementation. For example, investigators attempting to reduce PACU lengths of stay utilized the Monte Carlo simulation to quantify the impact of eliminating administrative delays [38]. In their study, 8.4% of patients experience an administrative delay leading to a prolonged PACU course (0.82 ± 0.58 hours vs. 0.29 ± 0.65 hours). Using baseline data, the authors created two hypothetical patient groups and simulated delayed and non-delayed PACU lengths of stay for 8.4% and 91.6% of patients, respectively. The Monte Carlo simulation allowed the investigators to predict the effect of their proposed intervention (elimination of administrative delays) to determine where resources should be dedicated based on the simulated effect (decreased PACU length of stay). Because the Monte Carlo simulation estimated total PACU time to decrease by less than 5% if administrative delays were eliminated, the authors sought alternative interventions with greater impacts. A similar method should be employed within the context of a graduate medical education program to maximize the impact of potential projects.

## Conclusions

QI projects must be performed to meet residency training goals. In order for these same projects to also meet organizational goals, advanced planning potentially using simulation may also be required. Because the most readily published QI projects are designed to improve trainee or provider knowledge, these projects are unlikely to meet organizational quality goals, by themselves.
